# Abiotic Stress Tolerance of Coastal Accessions of a Promising Forage Species, *Trifolium fragiferum*

**DOI:** 10.3390/plants10081552

**Published:** 2021-07-28

**Authors:** Una Andersone-Ozola, Astra Jēkabsone, Līva Purmale, Māris Romanovs, Gederts Ievinsh

**Affiliations:** Department of Plant Physiology, Faculty of Biology, University of Latvia, 1 Jelgavas Str., LV-1004 Rīga, Latvia; una.andersone-ozola@lu.lv (U.A.-O.); astra.jekabsone@lu.lv (A.J.); liva.purmale@lu.lv (L.P.); maris.romanovs@lu.lv (M.R.)

**Keywords:** crop wild relatives, cutting, forage legume, soil moisture, strawberry clover, trampling

## Abstract

Crop wild relatives are valuable as a genetic resource to develop new crop cultivars, better adapted to increasing environmental heterogeneity and being able to give high quality yields in a changing climate. The aim of the study was to evaluate the tolerance of different accessions of a crop wild relative, *Trifolium fragiferum* L., from coastal habitats of the Baltic Sea to three abiotic factors (increased soil moisture, trampling, cutting) in controlled conditions. Seeds from four accessions of *T. fragiferum,* collected in the wild, were used for experiments, and cv. ‘Palestine’ was used as a reference genotype. Plants were cultivated in asymbiotic conditions of soil culture. Treatments were performed in a quantifiable way, with three gradations for soil moisture (optimum, waterlogged, flooded) and four gradations for both trampling and cutting. All accessions had relatively high tolerance against increased soil moisture, trampling, and cutting, but significant accession-specific differences in tolerance to individual factors were clearly evident, indicating that the studied wild accessions represented different ecotypes of the species. Several wild accessions of *T. fragiferum* showed stress tolerance-related features superior to these of cv. ‘Palestine’, but TF1 was the most tolerant accession, with a very high score against both waterlogging and cutting, and a high score against trampling.

## 1. Introduction

From the view of climate change and growing anthropogenic impact on ecosystems, increasing the sustainability of agricultural production is of critical importance. In order to ensure productivity and increased added value in this complicated situation, it is necessary to develop new, more diverse crop cultivars, better adapted to increasing environmental heterogeneity and being able to give high quality yields in conditions of changing climate [1]. Crop wild relatives (CWR) are valuable as a genetic resource in this respect, representing a source of environmental tolerance-associated characteristics [2,3,4].

According to the current targets of the Convention on Biological Diversity, national inventories of CRWs need to be performed in order to identify necessary conservation measures [5]. In Latvia, CWRs are relatively poorly represented, with only perennial forage grasses and legumes being more widely accessible. Legumes are especially important, from the view of agricultural sustainability, both as high quality protein crops, and as nitrogen-fixing species, which lead to increased soil fertility without a need to apply high doses of mineral nitrogen fertilizers [6]. Scientific attention has been focused recently on wild genetic resources of several traditional clover (*Trifolium*) species, in Latvia and other countries of the region, for their potential use as forage crops [7,8,9,10,11]; however, one extremely rare wild clover species, *Trifolium fragiferum* L., has remained neglected [12]. While not commercially used in Europe, *T. fragiferum* has been cultivated in other regions of the world, for example, Australia, New Zealand, USA [13,14].

The natural range of distribution of *T. fragiferum* is connected with Eurosiberian and Mediterranean centers of plant diversity [15]. In Northern Europe *T. fragiferum* is relatively rare and is exclusively associated with an endangered habitat, “Baltic coastal meadow” (A2.5b) [16]. Morphologically, *T. fragiferum* represents a perennial semi-rosette plant with proliferating creeping basal shoots [17]. An ability of monopodially branching shoots (stolons)—to form roots at the nodes at high moisture—provides high clonal reproduction potential [18]. There is scientific evidence available that wild accessions of *T. fragiferum* are exceptionally potential genetic resource in potential the improvement of forage quality and sustainability within a broader geographical perspective, including Northern Europe. 

*T. fragiferum* has moderate-to-good salinity tolerance and has potential for use in pastures and meadows with salt problems, in contrast to the similar species *Trifolium repens* [19]. *T. fragiferum* is also characterized as relatively tolerant to moderate soil salinity [15], it is sometimes classified as mesohydrohalophilic euhalophyte [20]. Moreover, the species has considerable tolerance to other soil-related problems, such as alkalinity and flooding [15]. Previous studies have shown comparatively good flooding tolerance in *T. fragiferum* [21,22]; it was shown to be 20 days, which is among the highest between the legume species, being in line with *T. repens* and *Lotus corniculatus* [23]. A genus-wide analysis of salinity and waterlogging tolerance of *Trifolium* species has been performed, showing that *T. fragiferum* was among the seven species least affected by hypoxic soil conditions [24]. Characteristic clonal (stoloniferous) growth habit of *T. fragiferum* supports extreme tolerance to close continuous grazing [25]. In addition, *T. fragiferum* can be expected to have good trampling tolerance similar to that of other *Trifolium* species [26].

The aim of the present study was to evaluate abiotic stress tolerance (soil moisture, trampling, cutting) of several wild accessions of *T. fragiferum,* from coastal habitats of the Baltic Sea, in controlled conditions. It was especially asked whether these accessions have tolerance characteristics comparable to, or even better than, those of widely used commercial elite cultivars of *T. fragiferum*.

## 2. Results

### 2.1. Effect of Increased Soil Moisture

**Figure 1:**
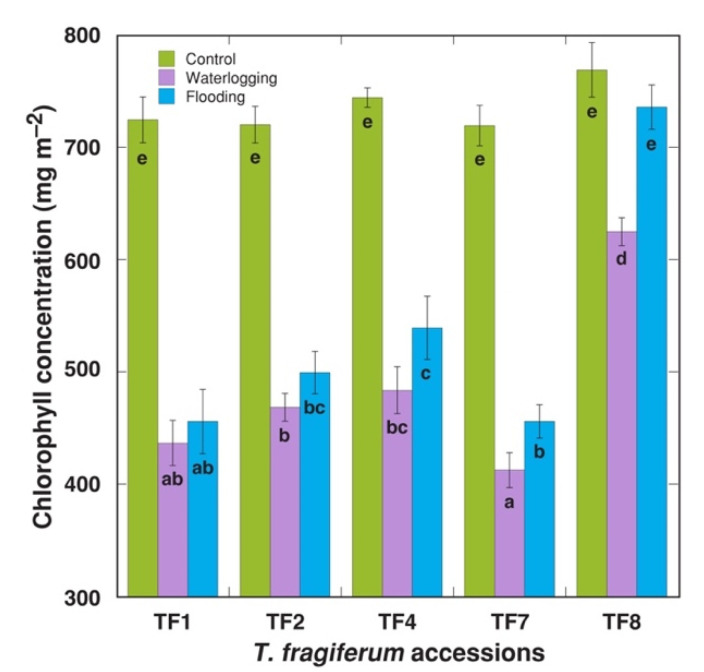
Direct effect of increased soil moisture in the form of waterlogging and flooding on leaf chlorophyll concentration of *Trifolium fragiferum* plants of different accessions. Measurements were performed after three weeks of treatment. Data are means ± SE from five plants, with three independent measurements each. Different letters indicate statistically significant differences *(p* < 0.05).

**Figure 2:**
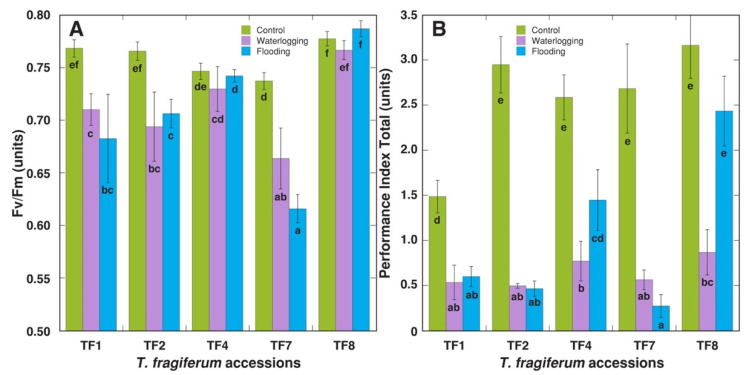
Direct effect of increased soil moisture in the form of waterlogging and flooding on chlorophyll *a* fluorescence parameters Fv/Fm (**A**) and performance index total (**B**) of *Trifolium fragiferum* plants of different accessions. Measurements were performed after three weeks of treatment. Data are means ± SE from five plants, with three independent measurements each. Different letters indicate statistically significant differences (*p* < 0.05).

Morphologically, elongation of leaf petioles was the first characteristic direct response of *T. fragiferum* plants in flooded conditions. Loss of chlorophyll in leaves with progression of treatment duration was another visual response; therefore, both chlorophyll concentration and chlorophyll *a* fluorescence were measured immediately after the end of the treatment. Decreased leaf chlorophyll concentration was evident in both waterlogged and flooded plants, but waterlogging tended to have a more severe effect, with statistically significant differences between the two treatments for TF7 and TF8 (Figure 1). The lowest negative effect of increased soil moisture on leaf chlorophyll concentration was seen for TF8, where there was no significant decrease of the concentration for flooded plants. Maximum quantum efficiency of photochemistry of Photosystem II, Fv/Fm, significantly decreased by increased moisture in TF1, TF2, and TF7, but no effect was evident for TF4 or TF8 (Figure 2A). Similarly, the indicator of photochemical performance of photosynthesis, performance index total, tended to decrease by both treatments, but the effect was less pronounced for TF4 and TF8, with no significant decrease in the parameter for flooded TF8 plants (Figure 2B).

**Figure 3:**
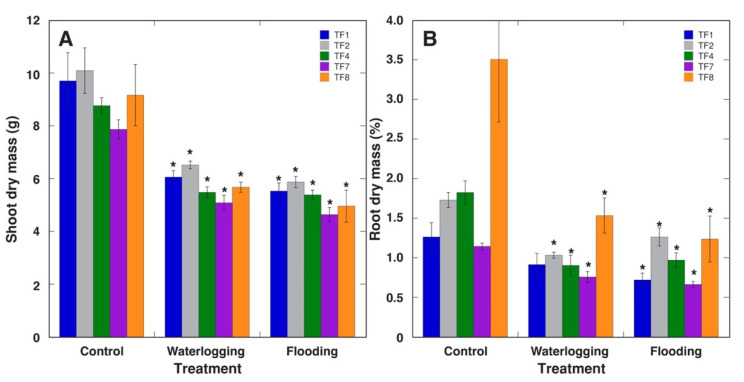
Effect of increased soil moisture in the form of waterlogging and flooding on shoot dry mass (**A**) and root dry mass (**B**) of *Trifolium fragiferum* plants of different accessions. Measurements were performed after three weeks of treatment plus four weeks of recovery. Data are means ± SE from five plants. Asterisks indicate statistically significant difference (*p* < 0.05) from control for the respective accession.

Four weeks after the termination of a three-week-long treatment, dry mass of aboveground parts was significantly lower for *T. fragiferum* plants, under both waterlogging and flooding, for all accessions (Figure 3A). Dry mass of roots also decreased, but the effect was not significant for waterlogged TF1 plants (Figure 3B).

Waterlogging and flooding clearly had different effects on morphological parameters and biomass of various plant parts (Table 1). Thus, waterlogging significantly reduced stolon number for TF4 and TF7, whereas flooding reduced this parameter for TF1 and TF7. Growth of stolons was the most sensitive to increased substrate moisture, as both waterlogging and flooding reduced both total length and dry mass of stolons for all accessions of *T. fragiferum*. However, average stolon length was significantly inhibited only for TF1 and TF2 by both treatments, and for TF8 by flooding. Number of leaves was not significantly affected by increased moisture for TF8, but it was decreased by flooding for TF1, TF2, and TF7, and by waterlogging for TF4. Biomass of leaf petioles was affected relatively little by increased moisture, with significant reduction by waterlogging for TF2, TF4, and TF8, and by flooding for TF7. All accessions except TF1 showed significant reduction of leaf blade biomass by both treatments. For TF1, only flooding had significant effect of this parameter.

**Figure 4:**
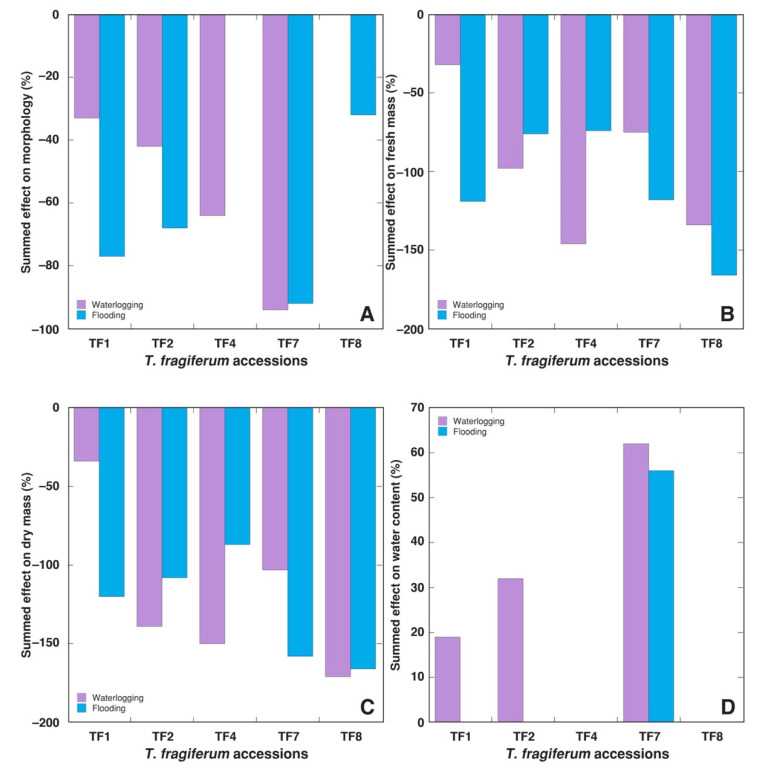
Summed effect of increased soil moisture in the form of waterlogging and flooding on morphology (**A**), fresh mass of plant parts (**B**), dry mass of plant parts (**C**), and water content in plant parts (**D**) of *Trifolium fragiferum* plants of different accessions. Measurements were performed after three weeks of treatment plus four weeks of recovery. Only statistically significant effects are taken into the account.

To compare the relative effect of increased moisture treatments on different *T. fragiferum* accessions, the percentage change from controls for the various parameters by the respective treatment was summed by parameter group, taking into the account only statistically significant changes. This comparison allowed us to notice that morphological parameters (number of stolons, number of leaves, average and total length of stolons) were the least affected by increased moisture (Figure 4A). In this respect, flooding was more unfavorable than waterlogging for TF1, TF2, and TF8, but the opposite effect was evident for TF4. Effect of increased moisture on fresh (Figure 4B) and dry (Figure 4C) mass of plant parts was relatively similar: for TF1 and TF7 flooding was more unfavorable in comparison to flooding, but for TF2 and TF4 the effect was reversed. For TF8, fresh mass was more negatively affected by flooding, but identical effect was evident for dry mass. Waterlogging resulted in significantly increased tissue water content in TF1, TF2, and TF7, but in the case of flooding only TF7 was affected in this respect (Figure 4D).

### 2.2. Effect of Trampling

**Figure 5:**
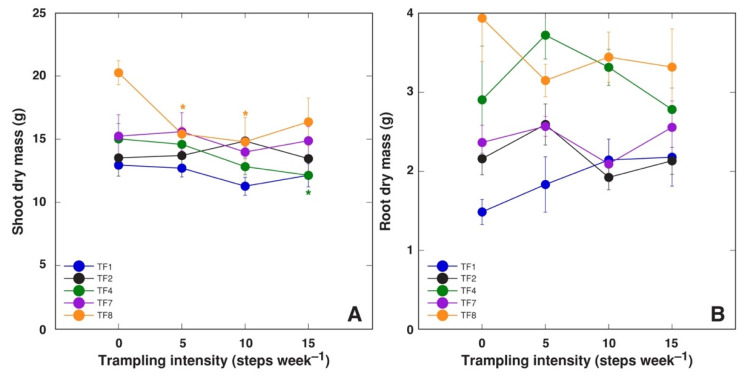
Effect of increasing trampling intensity on shoot dry mass (**A**) and root dry mass (**B**) of *Trifolium fragiferum* plants of different accessions. Measurements were performed after five weeks of treatment plus three weeks of recovery. Data are means ± SE from five plants. Asterisks indicate statistically significant difference (*p* < 0.05) from control for the respective accession.

Dry mass of shoots was negatively affected by trampling only for TF4 at the highest intensity and TF8 at 5 and 10 steps week^–1^ (Figure 5A). Root dry mass was not significantly affected at any trampling intensity for all accessions, but this parameter tended to increase with increasing trampling intensity for TF1 (Figure 5B).

Number of stolons and number of leaves were not negatively affected by trampling, these parameters even significantly increased for TF1 (number of stolons at trampling intensity 15 times week^–1^, number of leaves at trampling intensity 5 times week^–1^) and TF4 (number of leaves at trampling intensity 5 times week^–1^) (Table 2). Also, dry mass of leaf petioles significantly increased for TF2 at the two lower trampling intensities. However, a statistically significant negative effect was evident in the case of average stolon length for TF1 and TF4 (at the two highest trampling intensities) and for TF8 (at all three trampling intensities). Dry mass of stolons significantly decreased for TF1 (treatment 10 times week^–1^), TF4 (15 times week^–1^), and TF8 (5 times week^–1^). In addition dry mass of petioles significantly decreased for TF4 at the highest trampling intensity. Water content in different plant parts was not significantly affected by trampling (data not shown).

**Figure 6:**
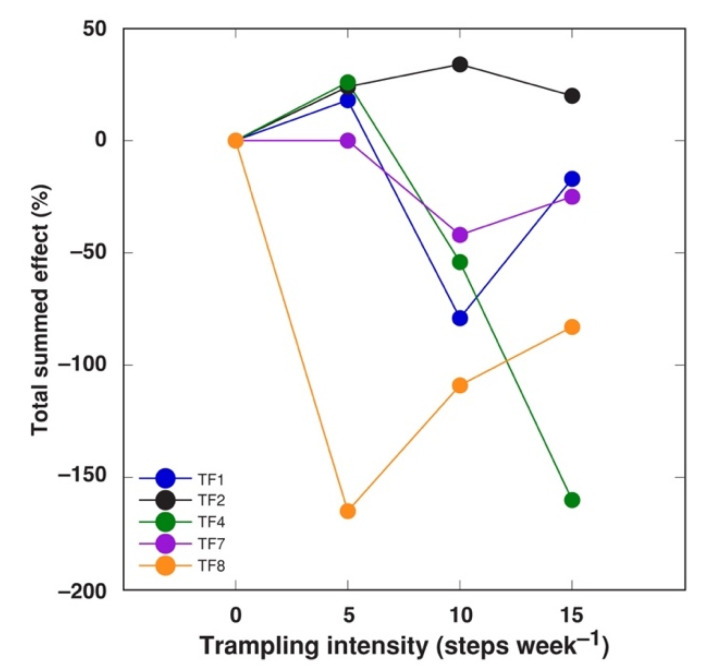
Total summed effect of increasing trampling intensity on morphology and biomass of *Trifolium fragiferum* plants of different accessions. Measurements were performed after five weeks of treatment plus three weeks of recovery. Only statistically significant effects are taken into the account.

When total summed effect of trampling was evaluated, it appeared that, at the lowest intensity, there was a positive total summed effect on TF1, TF2, and TF4 (Figure 6). Further, only accession TF2 showed a positive effect by trampling at all intensities. Only TF4 responded to trampling in an intensity-dependent manner, but, for TF1 and TF7, there was a tendency for recovery at the highest trampling intensity. TF8 was extremely sensitive to the lowest trampling intensity treatment, with pronounced recovery with further increase in trampling intensity.

### 2.3. Effect of Cutting

**Figure 7:**
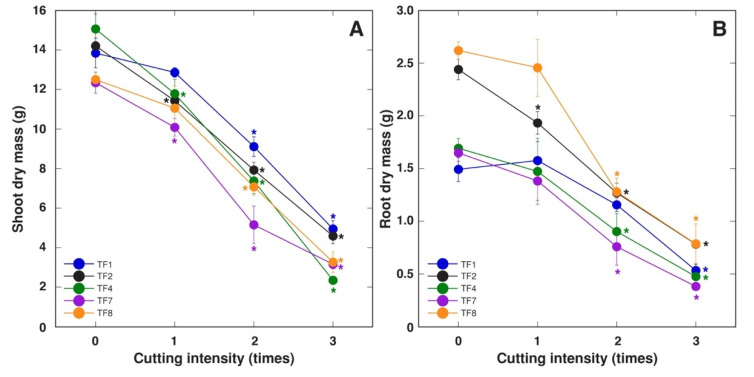
Effect of increasing cutting intensity on shoot dry mass (**A**) and root dry mass (**B**) of *Trifolium fragiferum* plants of different accessions. Data are means ± SE from five plants. Measurements were performed after six weeks of treatment plus three weeks of recovery. Asterisks indicate statistically significant difference (*p* < 0.05) from control for the respective accession.

A single cutting episode had no significant effect on biomass of aboveground parts only for *T. fragiferum* accessions TF1 and TF8, with further significant decrease for all accessions with increasing cutting intensity (Figure 7A). Root growth was less sensitive to cutting, with significant decrease at single cutting only for TF2, and TF1 was the most resistant in this respect (Figure 7B).

Number of stolons was the parameter least sensitive to cutting, but stolon growth and leaf growth was affected for all accessions, though, to different extents (Table 3). Thus, TF2 showed significant reduction of these parameters by all cutting intensities, but for TF7 leaf growth was not significantly affected by single cutting. For TF4, single cutting did not affect stolon and leaf growth, it even significantly stimulated appearance of new stolons. Accessions TF8 and TF1 were the most resistant in respect to leaf and stolon growth.

**Figure 8:**
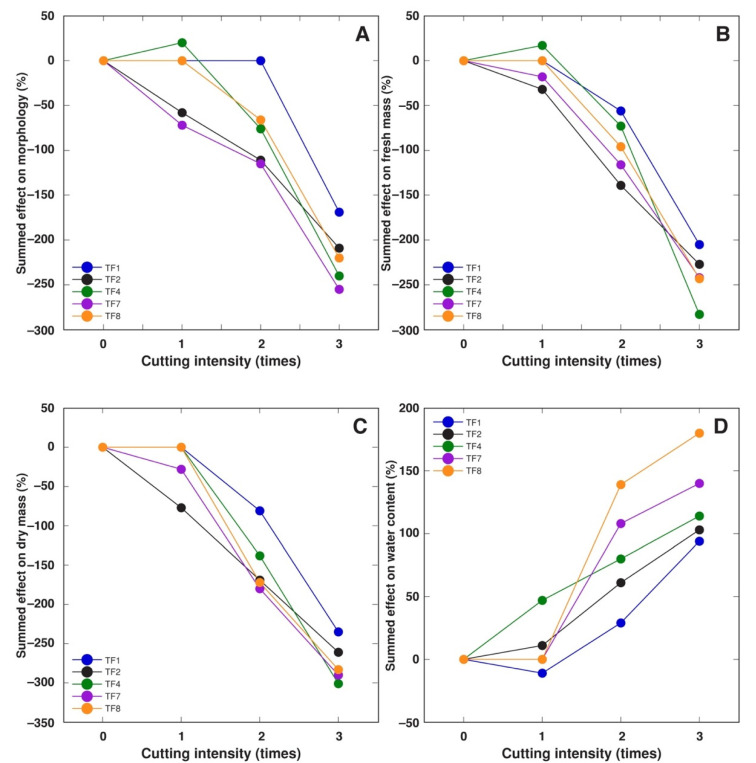
Summed effect of increasing cutting intensity on morphology (**A**), fresh mass of plant parts (**B**), dry mass of plant parts (**C**), and water content in plant parts (**D**) of *Trifolium fragiferum* plants of different accessions. Measurements were performed after six weeks of treatment plus three weeks of recovery. Only statistically significant effects are taken into the account.

These relationships were confirmed by analysis of summed effect of cutting, showing that, at the level of morphological parameters, TF1 had exceptional tolerance, followed by TF4 and TF8, but TF2 and TF7 were relatively susceptible (Figure 8A). The same order of tolerance was evident for summed effect on fresh mass (Figure 8B), and, to a lesser extent, dry mass of plant parts (Figure 8C). An interesting feature of cutting response was associated with a significant increase in water content in plant parts, which was the least pronounced in the most sensitive accession, TF1 (Figure 8D).

### 2.4. Average Abiotic Stress Tolerance of T. fragiferum Accessions

Relative comparison of overall tolerance of individual accessions of *T. fragiferum* to the tested abiotic factors by grading into four categories showed that TF1 was the most tolerant from these accessions, with very high score against both waterlogging and cutting, and high score against trampling (Table 4). Only flooding tolerance of TF1 was low. TF2 stood out with very high tolerance to trampling, but also had low tolerance to cutting, with moderate tolerance to increased soil moisture. TF7 had high tolerance to trampling, but scored low or moderate for other factors. The reference cultivar, TF8, appeared to be the most sensitive, with low scores for all factors but cutting.

**Figure 9:**
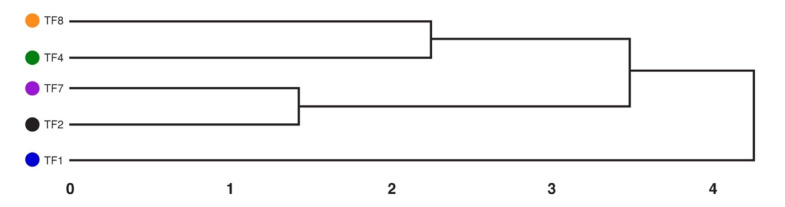
Cluster analysis of similarity in abiotic stress tolerance of *Trifolium fragiferum* plants of different accessions. Data are from Table 4. Euclidian distance with UPGMA clustering was used.

Cluster analysis confirmed the exceptional nature of TF1, being the most different from the other accessions (Figure 9). The highest degree of similarity was between accessions TF2 and TF7, but the least tolerant accession, TF8, was paired with TF4. Both accessions within each pair had the same degree of resistance to waterlogging and cutting (Table 4).

## 3. Discussion

In the present study, an asymbiotic cultivation system of *T. fragiferum* plants established from sterilized seeds in semi-sterile soil was used for comparison of abiotic stress tolerance between several wild accessions of the species. This setup allowed us to prevent possible genotype-specific differences in N_2_-fixing intensity arising from the spontaneous establishment of rhizobial symbiosis as a result of a random presence of bacteria in the cultivation substrate [27]. On the other hand, there is no doubt that presence of native rhizobial strains can affect the growth and physiological responses of legume species to various abiotic factors [28].

As it was initially expected, *T. fragiferum* accessions had relatively high tolerance against increased soil moisture, trampling, and cutting. However, significant accession-specific differences in tolerance to individual factors were clearly evident (Table 4) indicating that the studied wild accessions represented different ecotypes of the species. Most importantly, all accessions of *T. fragiferum* showed higher average tolerance in comparison to cv. ‘Palestine’, indicating that wild accessions can serve as potential donors of genes for abiotic stress tolerance. This finding supports the view that genetic diversity among *T. fragiferum* populations is sufficient to develop new cultivars with better performance in less favorable agroecological conditions [29]. The accession from a wet salt-affected meadow in city Liepāja (TF1) seemed to be especially promising in this respect, as it has very high reliability against waterlogging and cutting and high endurance against trampling (Table 4).

The waterlogging tolerance of various *Trifolium* species has been associated with extensive development of lateral roots with high porosity [24,30]. Due to better aeration of newly-formed lateral roots, oxygen flux from atmosphere to roots increases, forming a morphological basis of tolerance to high soil moisture. A four-week treatment in hypoxic conditions reduced biomass of *T. fragiferum* cv. ‘Palestine’ shoots by 22 to 26% and that of roots by 10 to 24%, together with more than a two-fold increase of root porosity, positioning the species among the seven most waterlogging-tolerant *Trifolium* species [24]. In the present experiments, shoot biomass was reduced for TF8 (cv. ‘Palestine’) by 38% and that of roots by 56% as a result of waterlogging, but flooding resulted in even larger biomass reduction (by 46 and 65%, for shoots and roots, respectively). However, all wild accessions of *T. fragiferum* had better tolerance to hypoxic conditions.

It is important to note that resistance to high soil moisture and recovery ability, after moisture episodes, can be differentially expressed traits [26]. Among typical grassland species, grasses are more resistant to flooding in comparison to legumes, but legumes have better recovery ability after flooding episodes [31]. Accession TF8 in the present study had the highest immediate resistance to soil moisture, but this accession had relatively low recovery ability, resulting in low overall tolerance (Table 4). Similarly, growth of plants in waterlogged-only soil vs flooded soil can be differentially affected. This was the case also in the present study where the accessions TF1 and TF7 were more tolerant to waterlogging in comparison to flooding, TF2 and TF8 were equally affected, but TF4 was more tolerant to flooding than to waterlogging (Table 4).

Leaf yellowing (loss of chlorophyll) has been indicated as an excellent indicator of flooding damage in legume species [23]. However, leaf N concentration was only slightly reduced by flooding in *T. fragiferum* [21]. According to relatively small negative changes in leaf chlorophyll concentration (Figure 2) and chlorophyll fluorescence parameters (Figure 3), TF8 was highly resistant during an acute episode of increased moisture, but had lower recovery potential after the episode, especially at the level of biomass accumulation, but morphological parameters were relatively unaffected (Figure 4).

Only a limited number of experimental studies aiming at assessing trampling effects on plants has been performed in controlled conditions. It has been argued that studies of trampling effects in controlled conditions, while allowing for measurement of plant growth responses to quantifiable intensity of trampling, have several limitations, such as the inability of a mechanical foot to fully reproduce human or animal trampling [32]. Both soil compaction and shoot damage of plants are the main plant growth-affecting factors resulting from trampling. It is also indicated that these two factors may have contradictory effects [32]. Soil compaction, itself, results in increased mechanical resistance to root growth, often resulting in root growth inhibition [33,34]. Mechanical damage due to trampling is often associated with stress ethylene-mediated inhibition of elongation growth [35]. Root growth was not significantly affected at any trampling intensity for any *T. fragiferum* accession (Figure 5B), showing exceptional ability of the species to grow in compacted soil. Root biomass of the highly tolerant accession TF1 even tended to increase with trampling intensity. However, stolon length decreased for all *T. fragiferum* accessions, except the most tolerant TF2, and was a partially trampling intensity-dependent phenomenon (Table 3).

Prostrate growth form, as in the case of wild accessions of *T. fragiferum*, is often associated with high resistance to trampling [32]. In contrast, cv. ‘Palestine’ and several other commercial cultivars of *T. fragiferum* have a more erect appearance [36], which is clearly associated with the relatively lower trampling tolerance of cv. ‘Palestine’ found in the present study (Table 5). Additional morphological features often associated with trampling-related mechanical resistance include stem flexibility, small and thick leaves, and flexible leaf petioles etc. [37], but these characteristics were not assessed in the present study.

Cutting and grazing tolerance are extremely important characteristic of forage plant species, as grassland management includes repetitive removal of plant biomass either by mowing or ruminant grazing [38]. *Trifolium* species usually show significant variation in respect to grazing tolerance in field conditions, with prostrate-growing *T. repens* having better persistence in comparison to the more erect *Trifolium pratense* [39]. Within a single species, as in the case of relatively grazing-tolerant *T. repens*, large leaved varieties showed higher yields under rotational grazing and cutting management [40]. However, it has been noted that different plant characteristics can be important for cutting vs grazing resistance, as grazing by ruminant animals results in a different type of damage to stolons in comparison to simple cutting [41]. Leaf size of *T. fragiferum* accessions was not evaluated in the present study, but indirect evidence based on comparison of dry mass of single leaf blade suggested that TF8, with the largest leaf mass (0.014 g), had the same cutting tolerance as TF4, with the lowest leaf mass (0.009 g).

Good tolerance of *T. fragiferum* to cutting seems to be associated with a presence of large number of dormant, low-placed, above-ground meristems, which development is induced by removal of apical meristems. In this species, each leaf axil bears a meristem able to develop either lateral stolon or inflorescence [18]. Therefore, after cutting, remaining nodes are able to quickly develop new fast-elongating stolons, with leaves using root-stored reserves, leading to fast reestablishment of photosynthetic function.

In the present study, only effects of different single factors were evaluated. However, interactions between these factors could be proposed in the case of simultaneous exposure to several of them. For example, for a typical temperate grassland legume species, such as *T. repens*, high plant abundance has resulted from interaction of defoliation and trampling [42]. In addition, other abiotic environmental factors as well as symbiotic relationshipsm both with nitrogen-fixing rhizobacteria and mycorrhizal fungi could be important as determinants for overall resilience and biomass production of *T. fragiferum* as a perennial legume species, as can be proposed from results of studies in natural conditions [43].

In general, morphological parameters (number of stolons and leaves) were less sensitive proxies to unfavorable abiotic factors in comparison to biomass accumulation in different plant parts. It is evident that morphological indices could reflect development-related alterations, which in general are less sensitive to changes in abiotic factors in comparison to plant growth. In addition, tissue water content in plant parts showed significant changes in some accessions of *T. fragiferum* subjected to increased soil moisture (Figure 4D), as well as in a result of foliage cutting (Figure 8D). In the latter case, cutting forced plants to produce new foliage structures, and plants of equal age but with higher cutting intensity had larger proportion of younger tissues. It has been noted that leaves’ water content negatively correlates with their age, at least for several plant species [44].

## 4. Materials and Methods

### 4.1. Plant Material and Experimental Setup

Seeds from four accessions of *T. fragiferum,* collected in the wild (TF1, Liepāja; TF2, Jūrmala/Lielupe; TF4 Rīga/Skanste; TF7, Ainaži) and stored at 4 °C were used for the establishment of plants for experiments (Table 5). *T. fragiferum* cv. ‘Palestine’ (TF8), seeds obtained from Sheffield’s Seeds Company (Locke, NY, USA), was used as a reference genotype. Plants were cultivated in asymbiotic conditions of soil culture in an automated greenhouse. No symbiotic nodules were seen on roots at the termination of the experiments. Five individual plants per accession per treatment were used. Treatments were performed in a quantifiable way, with three gradations for soil moisture and four gradations for both trampling and cutting.

### 4.2. Plant Propagation and Establishment of Experimental Material

Seeds were surface-sterilized with a half-diluted commercial bleach (ACE, Procter & Gamble, Warszawa, Poland), containing 5% sodium hypochlorite, for 10 min, followed by three rinses with deionized water (10 min each). Seeds from wild populations were scarified under binocular loupe with a scalpel after imbibing in deionized water for 48 h. Prepared seeds were placed in 1 L plastic plant tissue culture containers, filled with autoclaved (1 atm, 20 min) garden soil (Biolan, Eura, Finland) and closed with lids, and further cultivated for two weeks in a growth cabinet (light/dark period of 16/8 h, photosynthetically active radiation with a photon flux density 100 µmol m^–2^ s^–1^, day/night temperature 15/20 °C). After the appearance of the first two true leaves, seedlings were individually transplanted to 250 mL plastic containers filled with a mixture of quartz sand (Saulkalne S, Saulkalne, Latvia) and heat-treated (60 °C, 24 h) garden soil (Biolan, Eura, Finland) 1:5 (*v*/*v*). Containers were placed in 48 L plastic boxes, closed with lids, in an experimental automated greenhouse (HortiMaX, Maasdijk, Netherlands) with supplemented light from Master SON-TPIA Green Power CG T 400 W (Philips, Amsterdam, Netherlands) and Powerstar HQI-BT 400 W/D PRO (Osram, Munich, Germany) lamps (380 µmol m^–2^ s^–1^ at the plant level) for a 16 h photoperiod, with day/night temperature 24/16 °C, and relative air humidity of 60 to 70%. Boxes were periodically ventilated to acclimate seedlings to greenhouse conditions. Two weeks later, seedlings were individually transplanted to 1.3 L plastic containers, filled with a mixture of quartz sand (Saulkalne S, Saulkalne, Latvia) and heat-treated (60 °C, 24 h) garden soil (Biolan, Eura, Finland) 1:3 (*v*/*v*). Experiments were started after a week-long period of acclimatization in a greenhouse.

During all experiments, plants were kept in a greenhouse in the same conditions as indicated above and irrigated with deionized water every other day. Substrate water content was monitored with HH2 moisture meter equipped with WET-2 sensor (Delta-T Devices, Burwell, UK) and kept at 50 to 60%, except those plants in the substrate moisture experiment. Every other week, plants were fertilized with Yara Tera Kristalon Red and Yara Tera Calcinit fertilizers (Yara International, Oslo, Norway), except those plants in the substrate moisture experiment, which were not fertilized during the three weeks of treatment. A stock solution was prepared for each fertilizer (100 g L^–1^) and the working solution contained 25 mL of each per 10 L deionized water, used with a rate 100 mL per container. Individual containers were randomly redistributed weekly on a greenhouse bench.

### 4.3. Increased Soil Moisture

The effect of increased substrate moisture was evaluated, either by keeping plants in waterlogged condition or by flooding with water above substrate level. For treatments, containers with plants were placed inside larger plastic containers (4.5 L) filled with 1.5 L deionized water (waterlogging) or filled with deionized water 2 to 3 cm above substrate level (flooding). Both treatments resulted in 80 to 85% substrate moisture. Weights (about 0.5 kg per container) were used to ensure stability of containers with plants in the case of flooding treatment. Control plants were maintained at 50 to 60% substrate moisture. Different moisture regimes were maintained for three weeks, followed by four weeks of recovery. Leaf chlorophyll concentration and chlorophyll *a* fluorescence were measured nondestructively after termination of treatments, as described below.

### 4.4. Trampling

Before treatment, foliage of greenhouse-acclimated plants was cut to 5 cm height and allowed to regrow for one week. Quantifiable trampling treatment was performed using a custom-built mechanical foot to simulate human trampling. The foot consisted of a metal cylinder filled with metal rods (total weight 10 kg), a cork-covered base in the form of the soil surface of a plant growth container, and a block system, allowing for easy and uniform impact from a height of 20 cm. Trampling was performed for five weeks with 5, 10, or 15 impacts per week, followed by three weeks of recovery. Only five trampling impacts per day were performed, repeated every other day for plants at medium and high trampling intensity. Plants were watered or fertilized only after trampling treatment to avoid additional effect of wet substrate. Untrampled plants were used as a control.

Treatment at the lowest trampling intensity (5 impacts week^–1^) resulted in 22.0% of soil compaction by volume, but further increase in trampling intensity resulted only in additional compaction by 4.5% (10 impacts week^–1^) and 1.7% (15 impacts week^–1^).

### 4.5. Cutting

Cutting was performed by scissors on straightened shoot 5 cm above substrate surface. Treatments involved control (no cutting), a single cutting episode, two cutting episodes, and three cutting episodes were performed every other week, for six weeks. Afterward, plants were allowed to recover for three weeks.

### 4.6. Measurements

Leaf chlorophyll concentration was measured for *T. fragiferum* plants in the substrate moisture experiment using a chlorophyll meter CCM-300 (Opti-Sciences, Hudson, NH, USA). Three fully grown actively photosynthesizing leaves per plant were measured on each of five plants per treatment per accession. Chlorophyll *a* fluorescence was measured, for plants in the substrate moisture experiment, in three leaves dark-adapted for at least 20 min by Handy PEA fluorometer (Hansatech Instruments, King’s Lynn, UK) on each of five plants per treatment per accession. Two fluorescence-derived parameters were calculated using PEA-Plus software (Hansatech Instruments, King’s Lynn, UK), namely, maximum quantum efficiency of Photosystem II, Fv/Fm, used as a general indicator of stress, and relative expression of photochemical performance, at four structural stages of electron transfer from water to NADPH, Performance Index Total [45].

At termination of the experiments, plants were individually separated in different parts. Number of inflorescences, stolons, and leaves was counted, and length of individual stolons was measured. Leaves were divided in petioles and blades and weighed separately, and fresh weight of stolons, flower stalks, inflorescences, and roots was determined. All individual parts were dried in a thermostat at 60 °C for 72 h and dry mass was measured. Water content in plant parts was calculated in g H_2_O per g dry mass.

### 4.7. Data Analysis

Flower-related characteristics showed extreme variability between individual plants at least for several accessions of *T. fragiferum*, therefore, these parameters were further used only for calculation of total shoot biomass per plant, but not as individual parameters.

The relative effect of different treatments were expressed as percent changes of the parameter in comparison to respective control plants. Comparison of the relative effect of treatments between different accessions was performed by means of summed percent changes, separately for morphological parameters (number of leaves and stolons, average and total length of stolons), fresh mass of separate plant parts, and dry mass of separate plant parts, as well as water content in plant parts. Total summed effect was calculated by combining percent effect on morphological parameters, fresh mass and dry mass. Only changes significantly statistically different from control values were taken into account for the calculation of summed effects.

Overall tolerance of the studied accessions was evaluated on the basis of the total summed effect for each abiotic factor separately. The particular range of summed effect for a respective factor was proportionally assigned to one of the following categories of tolerance, i.e., low (1), moderate (2), high (3), very high (4). The average value of an overall tolerance for a particular accession was calculated as a mean from the respective numeric values.

Results were analyzed and graphs were made by KaleidaGraph (v. 4.1, Synergy Software, Reading, PA, USA). Cluster analysis was performed using Euclidian distance with UPGMA clustering. Statistical significance of differences between all treatments was evaluated by one-way ANOVA minimum significant difference tests using a Microsoft Excel spreadsheet (www.biostathandbook.com/anova.xls accesssed on 15 July 2021) [46].

## 5. Conclusions

Wild accessions of *T. fragiferum* from salt-affected coastal habitats appear to be a promising source of environmental tolerance-associated characteristics. Each of all studied accessions had a unique physiological profile and had better overall abiotic stress tolerance in comparison with a standard *T. fragiferum* cultivar ‘Palestine’. However, additional information is necessary to fully evaluate the agrobiological potential of *T. fragiferum* accessions; as an example, that related to soil salinity and heavy metal tolerance. In addition, undergoing characterization of the genetic diversity of the accessions will allow to assess if any of them has a unique genetic profile in addition to its specific physiological type.

## Figures and Tables

**Table 1:** Effect of increased soil moisture on morphological parameters and dry mass of shoot parts of different *Trifolium fragiferum* accessions.

Table 1	No. of Stolons per Plant	Average Length of Stolons (mm)	Total Length of Stolons (cm)	DM of Stolons (g)	No. of Leaves per Plant	DM of Leaf Petioles (g)	DM of Leaf Blades (g)
TF1							
control	17.6 ± 1.6	322 ± 19	560 ± 41	3.23 ± 0.31	290 ± 30	1.46 ± 0.33	2.81 ± 0.55
WLG	15.8 ± 1.0	**216 ± 9**	**337 ± 14**	**2.02 ± 0.09**	254 ± 27	1.13 ± 0.14	2.06 ± 0.18
FLOOD	**14.0 ± 1.1**	**236 ± 13**	**322 ± 13**	**1.78 ± 0.03**	**204 ± 32**	1.05 ± 0.08	**1.89 ± 0.05**
TF2							
control	19.4 ± 3.3	337 ± 36	1193 ± 53	3.77 ± 0.32	264 ± 33	1.86 ± 0.27	3.30 ± 0.22
WLG	19.2 ± 0.7	**196 ± 6**	**721 ± 33**	**2.15 ± 0.08**	236 ± 6	**1.30 ± 0.06**	**2.50 ± 0.04**
FLOOD	15.0 ± 1.0	**227 ± 19**	**625 ± 73**	**1.77 ± 0.09**	**171 ± 7**	1.41 ± 0.13	**2.39 ± 0.11**
TF4							
control	13.0 ± 0.7	260 ± 17	333 ± 11	2.48 ± 0.11	157 ± 4	1.34 ± 0.08	2.37 ± 0.08
WLG	**9.0 ± 0.6**	230 ± 11	**205 ± 7**	**1.61 ± 0.09**	**106 ± 5**	**0.92 ± 0.02**	**1.57 ± 0.08**
FLOOD	10.2 ± 1.3	237 ± 18	**234 ± 15**	**1.88 ± 0.08**	136 ± 9	1.21 ± 0.08	**1.99 ± 0.04**
TF7							
control	14.6 ± 1.0	493 ± 38	713 ± 56	3.76 ± 0.19	231 ± 9	1.26 ± 0.08	2.38 ± 0.05
WLG	**8.0 ± 0.3**	377 ± 13	**302 ± 17**	**2.02 ± 0.11**	**170 ± 10**	1.04 ± 0.09	**1.80 ± 0.10**
FLOOD	**9.2 ± 1.0**	354 ± 29	**317 ± 21**	**1.78 ± 0.10**	**170 ± 7**	**0.89 ± 0.05**	**1.54 ± 0.08**
TF8							
control	12.8 ± 1.9	235 ± 33	294 ± 42	2.97 ± 0.52	188 ± 30	1.99 ± 0.42	4.08 ± 0.72
WLG	10.0 ± 0.6	203 ± 38	**196 ± 31**	**1.77 ± 0.14**	152 ± 13	**1.31 ± 0.08**	**2.46 ± 0.23**
FLOOD	9.8 ± 1.0	**159 ± 18**	**153 ± 18**	**1.50 ± 0.22**	132 ± 18	1.29 ± 0.20	**2.01 ± 0.31**

Measurements were performed after three weeks of treatment plus four weeks of recovery. WLG, waterlogging; FLOOD, flooding; DM, dry mass. Data are means ± SE per plant from five plants. Values in bold are statistically significantly different from control (*p* < 0.05).

**Table 2:** Effect of trampling intensity on morphological parameters and dry mass of shoot parts of different *Trifolium fragiferum* accessions.

Trampling Intensity (Steps Week^–1^)	No. of Stolons per Plant	Average Length of Stolons (mm)	Total Length of Stolons (cm)	DM of Stolons (g)	No. of Leaves per Plant	DM of Leaf Petioles (g)	DM of Leaf Blades (g)
TF1							
0	24.6 ± 0.9	275 ± 17	675 ± 36	4.47 ± 0.25	276 ± 11	1.78 ± 0.06	3.47 ± 0.28
5	28.2 ± 1.9	234 ± 11	660 ± 55	4.12 ± 0.31	**326 ± 11**	1.82 ± 0.10	3.67 ± 0.29
10	29.8 ± 2.7	**198 ± 15**	576 ± 26	**3.32 ± 0.09**	285 ±10	1.60 ± 0.21	3.66 ± 0.30
15	**40.0 ± 3.9**	**157 ± 10**	648 ±90	3.67 ± 0.49	385 ± 55	1.55 ± 0.15	3.45 ± 0.30
TF2							
0	34.6 ± 2.7	266 ± 20	943 ± 144	5.20 ± 0.97	483 ± 53	2.08 ± 0.04	4.34 ± 0.35
5	37.6 ± 1.3	229 ± 18	864 ± 88	5.02 ± 0.54	469 ± 38	**2.33 ± 0.06**	4.90 ± 0.23
10	36.8 ± 1.0	275 ± 16	1015 ± 74	6.03 ± 0.45	478 ± 15	**2.46 ± 0.07**	4.95 ± 0.08
15	32.6 ± 1.3	242 ± 10	788 ± 64	5.19 ± 0.48	443 ± 15	2.22 ± 0.18	4.41 ± 0.10
TF4							
0	32.2 ± 2.7	166 ± 9	535 ± 49	3.75 ± 0.38	319 ± 20	1.68 ± 0.12	3.27 ± 0.13
5	37.2 ± 3.8	137 ± 12	480 ± 30	3.49 ± 0.40	**401 ± 35**	1.76 ± 0.20	3.62 ± 0.31
10	36.2 ± 3.8	**123 ± 8**	410 ± 37	3.08 ± 0.51	331 ± 18	1.52 ± 0.09	3.06 ± 0.18
15	34.4 ± 0.8	**109 ± 4**	**374 ± 14**	**2.37 ± 0.15**	300 ± 8	1.34 ± 0.08	**2.47 ± 0.17**
TF7							
0	37.6 ± 3.0	432 ± 26	1611 ± 131	8.09 ± 0.55	360 ± 56	1.43 ± 0.20	4.03 ± 0.52
5	33.2 ± 3.6	406 ± 14	1335 ± 112	7.27 ± 0.56	411 ± 74	1.51 ± 0.19	4.18 ± 0.22
10	35.4 ± 4.7	362 ± 27	**1233 ± 79**	6.75 ± 0.20	387 ± 53	1.42 ± 0.08	4.01 ± 0.06
15	31.4 ± 2.7	383 ± 15	**1208 ± 133**	7.10 ± 0.56	341 ± 19	1.42 ± 0.18	4.33 ± 0.16
TF8							
0	25.8 ± 0.4	344 ± 34	892 ± 97	9.58 ± 0.70	380 ± 31	3.16 ± 0.26	6.35 ± 0.38
5	24.2 ± 1.2	**234 ± 32**	**634 ± 98**	**6.73 ± 0.61**	421 ± 45	2.54 ± 0.24	5.55 ± 0.38
10	29.4 ± 3.9	**198 ± 33**	**534 ± 88**	7.00 ± 1.28	466 ± 61	2.57 ± 0.32	5.19 ± 0.48
15	24.2 ± 1.8	**248 ± 27**	**610 ± 94**	8.41 ± 1.31	456 ± 50	2.77 ± 0.31	5.14 ± 0.78

Measurements were performed after five weeks of treatment plus three weeks of recovery. DM, dry mass. Data are means ± SE per plant from five plants. Values in bold are statistically significantly different from control (*p* < 0.05).

**Table 3:** Effect of cutting intensity on morphological parameters and dry mass of shoot parts of different *Trifolium fragiferum* accessions.

Cutting Intensity (times)	No. of Stolons per Plant	Average Length of Stolons (mm)	Total Length of Stolons (cm)	DM of Stolons (g)	No. of Leaves per Plant	DM of Leaf Petioles (g)	DM of Leaf Blades (g)
TF1							
0	40.4 ± 4.4	299 ± 22	1056 ± 108	4.48 ± 0.24	488 ± 49	2.71 ± 0.16	4.39 ± 0.48
1	39.2 ± 3.8	289 ± 20	1109 ± 64	4.58 ± 0.86	511 ± 55	2.29 ± 0.24	4.02 ± 0.17
2	34.0 ± 2.8	267 ± 25	891 ± 58	**3.25 ± 0.23**	355 ± 54	**2.06 ± 0.06**	**3.10 ± 0.13**
3	**20.2 ± 2.4**	235 ± 28	**474 ± 66**	**1.74 ± 0.20**	**177 ± 11**	**1.32 ± 0.13**	**1.79 ± 0.13**
TF2							
0	36.8 ± 2.6	360 ± 6	1321 ± 87	5.19 ± 0.25	554 ± 42	3.37 ± 0.10	4.72 ± 0.09
1	32.0 ± 0.9	**320 ± 9**	**1023 ± 37**	**3.95 ± 0.09**	**418 ± 14**	**2.89 ± 0.08**	**3.85 ± 0.09**
2	31.2 ± 2.6	**240 ± 7**	**749 ± 67**	**2.74 ± 0.31**	**358 ± 38**	**2.14 ± 0.11**	**2.89 ± 0.13**
3	**25.6 ± 2.3**	**165 ± 12**	**421 ± 51**	**1.42 ± 0.18**	**238 ± 16**	**1.47 ± 0.09**	**1.70 ± 0.13**
TF4							
0	29.8 ± 1.4	297 ± 12	880 ± 34	3.73 ± 0.21	338 ± 19	2.42 ± 0.13	3.19 ± 0.18
1	**35.8 ± 2.6**	250 ± 22	873 ± 38	3.39 ± 0.22	327 ± 8	2.41 ± 0.06	3.08 ± 0.15
2	28.4 ± 3.5	**233 ± 24**	**635 ± 53**	**2.43 ± 0.21**	**248 ± 23**	**1.86 ± 0.10**	**2.11 ± 0.17**
3	**20.0 ± 0.8**	**112 ± 6**	**226 ± 16**	**0.91 ± 0.06**	**112 ± 11**	**0.56 ± 0.05**	**0.75 ± 0.06**
TF7							
0	28.6 ± 1.3	478 ± 11	1370 ± 75	6.18 ± 0.45	345 ± 20	2.26 ± 0.10	3.75 ± 0.16
1	**23.4 ± 0.4**	**439 ± 5**	**1028 ± 19**	**4.49 ± 0.26**	**272 ± 10**	2.16 ± 0.14	3.28 ± 0.17
2	**23.4 ± 1.1**	**350 ± 9**	**818 ± 44**	**2.80 ± 0.25**	**221 ± 15**	**1.62 ± 0.11**	**2.25 ± 0.17**
3	**13.6 ± 0.7**	**210 ± 10**	**287 ± 26**	**1.10 ± 0.13**	**110 ± 4**	**0.86 ± 0.04**	**1.20 ± 0.07**
TF8							
0	20.0 ± 1.5	273 ± 26	545 ± 64	4.95 ±0.49	338 ± 44	2.53 ± 0.16	4.61 ± 0.42
1	23.6 ± 2.4	234 ± 27	540 ± 50	4.17 ± 0.46	351 ± 29	2.44 ± 0.29	4.05 ± 0.52
2	23.0 ± 1.7	**184 ± 14**	425 ± 48	**2.57 ± 0.24**	**223 ± 18**	**1.74 ± 0.16**	**2.66 ± 0.14**
3	**13.4 ± 1.8**	**115 ± 18**	**159 ± 36**	**1.02 ± 0.22**	**141 ± 25**	**0.95 ± 0.17**	**1.30 ± 0.17**

Measurements were performed after six weeks of treatment plus three weeks of recovery. DM, dry mass. Data are means ± SE per plant from five plants. Values in bold are statistically significantly different from control (*p* < 0.05).

**Table 4:** Relative comparison of overall tolerance of *Trifolium fragiferum* accessions to different abiotic factors.

Code	Waterlogging	Flooding	Trampling	Cutting	Average Tolerance
TF1	4	1	3	4	3.00
TF2	2	2	4	1	2.25
TF4	1	3	2	2	2.00
TF7	2	1	3	1	1.75
TF8	1	1	1	2	1.25

1, low; 2, moderate; 3, high; 4, very high.

**Table 5:** Accessions of *Trifolium fragiferum* from different locations in Latvia used in the present study.

Code	Location	Habitat	Coordinates	Year of Seed Collection
TF1	Liepāja	wet saline meadow	56°29′29″ N, 21°1′38″ E	2016
TF2	Jūrmala, Lielupe	saline river bank near estuary	57°0′11″ N, 23°55′56″ E	2016
TF4	Rīga, Skanste	degraded land in urban industrial area	56°57′46″ N, 24°7′2″ E	2020
TF7	Ainaži	dry coastal meadow	57°52′8″ N, 24°21′10″ E	2020
TF8	cv. ‘Palestine’	NA	NA	2020

## Data Availability

All data reported here is available from the authors upon request.
